# The Use of Ozenoxacin in Pediatric Patients: Clinical Evidence, Efficacy and Safety

**DOI:** 10.3389/fphar.2020.559708

**Published:** 2020-11-18

**Authors:** Giusy Davino, Tiziana D’Alvano, Susanna Esposito

**Affiliations:** Pediatric Clinic, Department of Medicine and Surgery, Pietro Barilla Children’s Hospital, University of Parma, Parma, Italy

**Keywords:** impetigo, methicillin-resistant *Staphylococcus aureus*, mupirocin-resistant *Staphylococcus aureus*, quinolone-resistant *Staphylococcus aureus*, ozenoxacin

## Abstract

Impetigo is the most common childhood skin infection in the world. There are two patterns of impetigo: *nonbullous* (or impetigo contagiosa) and *bullous*. The *nonbullous* type is due to *Staphylococcus aureus* and group A beta-haemolytic *Streptococcus* and occurs in 70% of impetigo cases. Impetigo is often a self-limited disease, but complications can sometimes occur. Therapy depends on the extent and site of the lesions and on the presence of systemic symptoms. The increase in multidrug resistance pathogens, such as methicillin-resistant *Staphylococcus aureus*, mupirocin-resistant *Staphylococcus aureus* or quinolone-resistant *Staphylococcus aureus*, requires the development of new antibiotics against these agents. The aim of this review is to evaluate the efficacy and safety of ozenoxacin in children compared to those of other approved topical antimicrobial therapies. The bactericidal activity against both susceptible and resistant organisms is a relevant feature of ozenoxacin because the bacterial strain and potential for resistance are generally not known at the beginning of therapy. Additionally, its minimal dermal absorption and its capability to reach high concentrations in the upper layers of the epidermidis agrees with the recommended practice aimed at avoiding the emergence of bacterial resistance in presence of a good safety profile. Further studies with real-life analyses and pharmacoeconomic evaluation are needed to confirm its role as first-line and second-line therapy in children with impetigo.

## Introduction

Impetigo is the most common childhood skin infection in the world (Bowen et al., 2015). There are two patterns of impetigo: *nonbullous* (or impetigo contagiosa) and *bullous* (Kliegman et al., 2011). The *nonbullous* type is due to *Staphylococcus aureus* and group A beta-haemolytic *Streptococcus* and occurs in 70% of impetigo cases. It is characterized initially by maculo-papular lesions and then by thin-walled vesicles that rupture, leaving superficial erosions covered by honey-coloured crusts that can involve both healthy and damaged skin (Hartman-Adams et al., 2014). The *bullous* type is due to toxin-producing *S. aureus* and is characterized by large, fragile vesicles and flaccid bullae on an erythematous base, which evolve into erosions with a thin, varnish-like crust; it is usually localized in intertriginous areas of the trunk and extremities (armpits, groins, between the fingers or toes, beneath the breasts) (Cole and Gazewood, 2007).

Impetigo is often a self-limited disease, but complications can sometimes occur. Acute poststreptococcal glomerulonephritis is a serious complication that could affect between 1 and 5% of patients with *nonbullous* impetigo (Brown et al., 2003). Potential complications of both *nonbullous* and *bullous* types are sepsis, osteomyelitis, arthritis, endocarditis, pneumonia, cellulitis, lymphadenitis, guttate psoriasis, toxic shock syndrome, and staphylococcal scalded skin syndrome (Mancini, 2000).

Therapy depends on the extent and site of the lesions and on the presence of systemic symptoms. The practical clinical recommendations suggest topical antibiotic therapy for localized lesions and systemic therapy with oral antibiotics in cases of extensive injury, failure or inability to perform topical therapy (Yamakawa et al., 2002; European Medicines Agency, 2014; Health Canada, 2017; U.S. Food and Drug Administration, 2017; Canton et al., 2018; González Borroto et al., 2018; López Cubillos et al., 2018; Tarragó et al., 2018; Vila et al., 2019; Torrelo et al., 2020). The increase in multidrug resistance pathogens, such as methicillin-resistant *Staphylococcus aureus* (MRSA), mupirocin-resistant *Staphylococcus aureus* or quinolone-resistant *Staphylococcus aureus*, requires the development of new antibiotics against these agents.

The aim of this review is to study the efficacy and safety of ozenoxacin in pediatric patients compared to those of other approved topical antimicrobial therapies. We searched for articles and papers on PubMed, Google Scholar, Clinicaltrials.gov, and Food and Drug Administration (FDA) and European Medicines Agency (EMA) websites, and we used “ozenoxacin,” “impetigo,” “topical antibiotics,” “retapamulin,” “mupirocin,” “fusidic acid,” “quinolone,” and “pediatric patients” as key words.

## Ozenoxacin

Ozenoxacin [1-cyclopropyl-8-methyl-7-(5-methyl-6-methylamino-pyridin-3-yl)-4-oxo-1,4-dihydro-quinoline-3-carboxylic acid] is a novel, nonfluorinated, topical quinolone. It is bactericidal against Gram-positive pathogens, including MRSA, MSSA, MRSE and *S. pyogenes*, and mupirocin-, and ciprofloxacin-resistant strains of *S. aureus* (Torrelo et al., 2020), inhibiting the enzymes DNA gyrase and topoisomerase IV, both of which are involved in bacterial DNA synthesis (Yamakawa et al., 2002). Its dual inhibitory activity against bacterial replication avoids the development of resistance (Vila et al., 2019); ozenoxacin also has a high accumulation inside Gram-positive bacterial cells, apparently due to its resistance to certain efflux pumps commonly found in *S. aureus* that affect other quinolones (López Cubillos et al., 2018). The absence of a fluorine atom in its molecular structure confers a better safety profile than other fluorinated quinolones, including a lack of quinolone-induced chondrotoxicity (González Borroto et al., 2018).

### Pharmacological Properties *in Vitro*

Ozenoxacin is effective as an antimicrobial agent against both staphylococci and streptococci, as demonstrated in comparative studies performed in 2010 and 2014 (Canton et al., 2018). A total of 1,097 clinical isolates were obtained from 49 centers located in the Czech Republic, Germany, the The Netherlands, Romania, South Africa, Spain and the United States during 2009–2010, and 1,031 other clinical isolates were obtained from January to December 2014 from 10 centers located in Argentina, Brazil, Colombia, Germany, Romania, South Africa, Spain, and Sweden and two sites in the United States. The antibacterial activity of ozenoxacin determined using MIC50 and MIC90 values was compared with that of 17 and 10 antimicrobial agents, respectively. These included the topical agents mupirocin, fusidic acid and retapamulin and other antimicrobials for a comparison of activity against resistant and susceptible strains. Ozenoxacin showed lower MICs against *S. aureus* isolates than fusidic acid, mupirocin, erythromycin, clindamycin, ciprofloxacin or levofloxacin, as established by the MIC90 level obtained from the studies examined. Additionally, against the levofloxacin-nonsusceptible *S. aureus* isolates, ozenoxacin proved to be the most powerful compound. Only clindamycin had an MIC50 equal to that of ozenoxacin. The other agents tested had higher MIC50 and MIC90 values than ozenoxacin. Study 1 showed that the bactericidal power of ozenoxacin against *S. pyogenes* isolates was comparable to that of retapamulin and that it had a twofold greater activity than mupirocin and 13 times greater activity than fusidic acid (Canton et al., 2018). In Study 2, ozenoxacin was the most potent agent tested against all *S. pyogenes* isolates, inhibiting 98.3% at an MIC of ≤0.03 mg/L (Canton et al., 2018). Ozenoxacin was 4-fold more active than erythromycin, clindamycin or retapamulin; at least eightfold more active than mupirocin; 64-fold more active than ciprofloxacin or levofloxacin; and at least 256-fold more active than fusidic acid.

### Pharmacological Properties *in Vivo*

In a mouse model of wound infection, using *S. aureus* as the infective agent, groups treated with ozenoxacin formulations and mupirocin 2% ointment and retapamulin 1% ointment, as well as between groups treated with different formulations of ozenoxacin 1% (one ointment, two gels and two creams) were compared (Tarragó et al., 2018). The most effective concentrations of ozenoxacin for reducing *S. aureus* counts after dermal application were 1 and 2%. The efficacy of ozenoxacin formulations was higher than that of retapamulin or mupirocin, especially with the selected final formulation, ozenoxacin 1% cream.

#### Authorization

In December 2017, the FDA approved the use of ozenoxacin 1% cream (10 mg/g) for the topical therapy of impetigo in adults, adolescents and children 2 months and older (U.S. Food and Drug Administration, 2017). Furthermore, pediatric investigation plans have been established by the EMA for ozenoxacin for the therapy of patients from 2 months to less than 18 years of age (European Medicines Agency, 2014). Trials are ongoing on oral ozenoxacin.

#### Posology

The approved posology for ozenoxacin is to apply a thin layer to the affected area twice daily for 5 days. The affected area should be no more than 100 cm^2^ in adults and pediatric patients 12 years of age and older, or more than 2% of the total body surface area in pediatric patients less than 12 years old. No dosage adjustments are necessary in patients with hepatic or renal impairments (Health Canada, 2017).

## Clinical Data on Ozenoxacin in Pediatric Patients

A phase I17 and two phase III studies (Gropper et al., 2014a; Rosen et al., 2018) of ozenoxacin for the therapy of impetigo were performed to evaluate its efficacy and safety profile by age group. The phase I study enrolled 38 patients aged ≥2 months to <18 years, of whom 36 completed the trial (Gropper et al., 2014b). The first phase III trial of ozenoxacin enrolled 335 patients aged ≥2 years to <18 years from Germany, Romania, South Africa, Ukraine, and the United States and compared treatments with ozenoxacin, placebo and retapamulin (as an internal validity control) in patients with impetigo (Gropper et al., 2014a). The second phase III trial of ozenoxacin enrolled 282 patients aged ≥2 months to <18 years from Germany, Romania, Russia, Spain, South Africa, and the United States and compared treatment with ozenoxacin and placebo in patients with a clinical diagnosis of impetigo (Rosen et al., 2018). For the analyses, data for the pediatric population were extracted, gathered and stratified into age groups: 2 to <6 months, 6 months to <2 years, 2 to <6 years, 6 to <12 years, and 12 to <18 years.

All studies had similar inclusion criteria: a total skin infection rating scale (SIRS) score of at least eight for the phase I trial and the first phase III trial and an SIRS score of at least three for the second phase III trial (including pus/exudate score of at least 1 in all the studies); a total area affected <100 cm^2^ and a total area affected <2% of the body surface area for the patients aged <12 years old (Gropper et al., 2014a; Gropper et al., 2014b; Rosen et al., 2018). The exclusion criteria were as follows: other concomitant underlying skin diseases, bacterial infections requiring systemic antibiotic therapy, immunodeficiency, and the use of other drugs that could confound the interpretation of the results. All studies used the same therapeutic schedule (i.e., topical application of ozenoxacin 1% cream or vehicle twice daily for 5 days). Furthermore, the results were assessed at regular intervals (before, during, at the end of therapy and at a follow-up visit). In all the studies, the visits included clinical evaluations to assess the progression or resolutions of the impetigo lesions, using the SIRS score, and taking blood and urine samples to evaluate the safety of the studies. Additionally, the phase I study included the plasma concentration of ozenoxacin (Gropper et al., 2014b), while both of the phase III trials included the collection of samples for microbiological investigations (Gropper et al., 2014a; Rosen et al., 2018). The primary efficacy endpoint of the studies examined was the clinical response (success or failure) at the end of the treatment (Gropper et al., 2014a; Gropper et al., 2014b; Rosen et al., 2018). Success was defined as a SIRS score of 0 for exudate/pus, crusts, positive thermotouch and pain and 0 or 1 for erythema, tissue oedema and itching or as improvement, defined as a >10% decrease in total SIRS score compared with baseline. In the first phase III trial, a randomized comparison of ozenoxacin, placebo and retapamulin showed that ozenoxacin is superior to placebo for clinical efficacy (Gropper et al., 2014a). In the intention-to-treat population, ozenoxacin was superior to placebo, and it was as effective as retapamulin (Gropper et al., 2014a). The superiority of ozenoxacin compared to placebo was also confirmed by the second randomized, double-blind, phase III study (Rosen et al., 2018). In the phase I study, 18 of 36 patients (50%) were labeled as cured (clinical success), and the other 50% had a clinical improvement (Gropper et al., 2014b).

Regarding microbiological efficacy, in all the trials the most isolated bacterial agent was *S. aureus*, followed by *S. pyogenes*; other isolated bacteria were *Staphylococcus epidermidis*, *Staphylococcus capitis* and *Staphylococcus hominis* (Gropper et al., 2014a; Gropper et al., 2014b; Rosen et al., 2018). The microbiological response after therapy with ozenoxacin vs placebo or retapamulin was evaluated at visit 2 (days 3–4 of therapy) and visit 3 (days 6–7 of therapy, end of therapy). Significantly higher microbiological success rates were achieved with ozenoxacin than with placebo in the overall combined population at visit 2 and visit 3 (Gropper et al., 2014a; Gropper et al., 2014b; Rosen et al., 2018). At visit 2, the microbiological success rates for ozenoxacin and placebo were 100 vs 60% for 0.5 to <2 years, 79.7 vs 59.2% for 2 to <6 years, 85.5 vs 55.4% for 6 to <12 years, and 83.3 vs 40.7% for 12 to <18 years. At visit 3, the microbiological success rates for ozenoxacin and placebo were 100 vs 60% for 0.5 to <2 years, 79.7 vs 63.5% for 2 to <6 years, 85.5 vs 64.8% for 6 to <12 years, and 83.3 vs 60.7% for 12 to <18 years. As shown in the first phase III trial, ozenoxacin was associated with a more rapid microbiological clearance than retapamulin, with a success rate of 70.8% vs 56.9% after only 3–4 days of treatment (Rosen et al., 2018).

In all the studies, 49 adverse events were registered in 38 patients (5.9%), all of which were mild 37) or moderate (12), and none were severe (Gropper et al., 2014a; Gropper et al., 2014b; Rosen et al., 2018). Ozenoxacin was well tolerated, and the reported adverse events were not related to its administration. Blood samples were gathered from the 38 pediatric patients of the phase I study. All ozenoxacin plasma samples were below the limit of quantification, defined as 0.5 ng/ml; therefore, the planned pharmacokinetic analyses were not performed. There were no clinically relevant changes in vital signs or blood and urinalysis tests.

Due to its bactericidal activity against both susceptible and resistant bacteria and its lack of quinolone-induced lesions in cartilage and bone, ozenoxacin could be an effective option, considering the restriction of the use of fluoroquinolones in the pediatric population to avoid damage to the musculoskeletal system.

### Ozenoxacin in Pediatric Clinical Practice

The data collected in the various preclinical and clinical studies show that ozenoxacin, a new nonfluorinated quinolone, developed for topical use, has bactericidal activity against several pathogens causing impetigo, including the multi-drug resistant ones, with a high efficacy and safety profile, thus representing a valid alternative for the therapy of impetigo in pediatric age (Gropper et al., 2014a; Gropper et al., 2014b; Rosen et al., 2018). These features were observed by collecting data for all patients aged <18 years old and divided into five age groups who participated in phase I or phase III trials. In these pediatric patients, clinical and microbiological success rates with ozenoxacin were superior to those with placebo.

The global spread of antibiotic resistance is an increasingly significant reality, and ozenoxacin is an important potential treatment option with an expanded spectrum against bacteria. Ozenoxacin was shown to be able to eradicate bacterial agents with both susceptible or resistant strains, and this is a notable feature because the resistance of a bacteria is generally not known at the beginning of the treatment. In the studies examined, approximately 80% of patients had a diagnosis of nonbullous impetigo (Gropper et al., 2014a; Gropper et al., 2014b; Rosen et al., 2018). The limited number of patients enrolled with bullous impetigo was insufficient to assess statistically significant data, and nonbullous impetigo was the only indication for treatment with 1% ozenoxacin cream, as specified on the data sheet. Although few patients under 6 months of age and with bullous impetigo were enrolled in phase III clinical trials of ozenoxacin, in the United States (Medimetriks Pharmaceuticals, Inc. 2017) and Canada (Health Canada, 2016), ozenoxacin 1% cream is indicated for topical treatment of nonbullous and bullous impetigo in patients aged 2 months and older.

## Conclusion

The bactericidal activity against both susceptible and resistant organisms is an important feature of ozenoxacin because the bacterial strain and potential for resistance are generally not known at the beginning of therapy. Additionally, its minimal dermal absorption and its capability to reach high concentrations in the upper layers of the epidermidis agrees with the current principles aimed at avoiding the emergence of bacterial resistance in presence of a good safety profile. Further studies with real-life analyses and pharmacoeconomic evaluation are needed to confirm its role as first-line and second-line therapy in children with impetigo and to evaluate its dermal absorption, especially in patients with chronic cutaneous diseases.

## Author Contributions

GD and TA performed the literature review and wrote the first draft of the manuscript. SE critically revised the text and made substantial scientific contributions. All authors approved the final version of the manuscript.

## Conflicts of Interest

The authors declare that the research was conducted in the absence of any commercial or financial relationships that could be construed as a potential conflict of interest.
